# Factors determining the most efficient spray distribution for marine cloud brightening

**DOI:** 10.1098/rsta.2014.0056

**Published:** 2014-12-28

**Authors:** P. J. Connolly, G. B. McFiggans, R. Wood, A. Tsiamis

**Affiliations:** 1 School of Earth, Atmospheric and Environmental Sciences, University of Manchester, Manchester M13 9PL, UK; 2 Department of Atmospheric Sciences, University of Washington, Seattle, WA 98195-160, USA; 3 Institute for Integrated Micro and Nano Systems, School of Engineering, University of Edinburgh, Edinburgh EH9 3JF, UK

**Keywords:** cloud brightening, aerosols, cloud-drops, albedo, geoengineering

## Abstract

We investigate the sensitivity of marine cloud brightening to the properties of the added salt particle distribution using a cloud parcel model, with an aim to address the question of, ‘what is the most efficient particle size distribution that will produce a desired cooling effect?’ We examine the effect that altering the aerosol particle size distribution has on the activation and growth of drops, i.e. the Twomey effect alone, and do not consider macrophysical cloud responses that may enhance or mitigate the Twomey effect. For all four spray generation methods considered, *Rayleigh jet*; *Taylor cone jet*; *supercritical fluid*; and *effervescent spray*, salt particles within the median dry diameter range *D*_m_=30–100 nm are the most effective range of sizes. The *Rayleigh jet* method is also the most energy efficient overall. We also find that care needs to be taken when using droplet activation parametrizations: for the concentrations considered, Aitken particles do not result in a decrease in the total albedo, as was found in a recent study, and such findings are likely to be a result of the parametrizations' inability to simulate the effect of swollen aerosol particles. Our findings suggest that interstitial aerosol particles play a role in controlling the albedo rather than just the activated cloud drops, which is an effect that the parametrization methods do not consider.

## 1. Introduction

The cost to society of increasing levels of carbon dioxide are likely to be significant [1]. Geoengineering, namely *deliberate large-scale manipulation of the planetary environment to counteract anthropogenic climate change* [2], has become a hotly contested topic in the atmospheric science community in the past few years (see [3]). The motivation for research into ‘geoengineering’ or ‘climate engineering’ is that it has the potential to *substantially reduce the costs and risks of climate change* (as Shepherd [2] concluded in a report by the UK's Royal Society).

Marine cloud brightening is a proposed geoengineering method to reduce the effects of global warming resulting from a changing climate [4,5]. The marine cloud brightening method involves using large numbers of ships to spray nebulized sea water into rising air below a marine boundary layer cloud. The resulting increased cloud condensation nucleus (CCN) concentrations mean that cloud droplet concentrations are increased, which has been hypothesized to result in clouds that are more reflective [6] and longer lasting [7]. For a recent review of the latest developments on this topic, see Latham *et al.* [8].

Research into this area of geoengineering has asked the question ‘what is the most efficient spray size that would result in a significant change to the cloud albedo?’ [8–10], without specifying what quantity the efficiency was being evaluated against. These former studies investigated the impact, on the change in cloud albedo, of adding sea spray of different sizes and at different particle *number concentrations*. However, central to optimizing the spray properties is the quantity we are trying to optimize with respect to. An obvious quantity to attempt to optimize is the amount of energy/power used to spray the aerosol into the atmosphere, which is the quantity we focus on herein.

At the time of writing, there are four main techniques of generating the sea spray that are possible candidates to consider in such a large-scale application (see [11,12]). These methods are referred to here as: (i) Rayleigh jet/jet instability; (ii) Taylor cone jets; (iii) supercritical fluids; and (iv) effervescent spray atomization. For an overview of these methods, see appendix A and references therein. Analysis of each of these spray techniques reveals that the power (electrical, mechanical or heat) that the spray techniques consume depends either on the flow rate of sea spray into the atmosphere, *Q* (in the case of the Taylor cone jet, super critical fluid and effervescent spray techniques), or on the product of flow rate and reciprocal of the aerosol median diameter, *D*_m_, (*a*/*D*_m_+*b*)*Q* (in the case of the Rayleigh jet-instability method). Hence, in our paper, we address the question of what are the optimal spray particle parameters: (i) for a given mass of sea water sprayed, *Q*, and (ii) for the parameter *χ*=(*a*/*D*_m_+*b*)*Q*. We show in the appendices that these two parameters should approximately scale with the amount of energy that either of the proposed spray methods use (see appendices for details).

We also address the use of *physically based parametrization schemes* for determining the optimal spray parameters. Our aim here is to investigate in detail an effect shown by Alterskjaer & Kristjánsson [10], where very small injected aerosol particles suppressed the cloud albedo, rather than enhanced it. Recently, Alterskjaer and Kristjánsson investigated the effect of injecting sea spray into marine boundary layer clouds in a global model. A physically based parametrization was used to determine how many cloud droplets would form on a combination of background aerosol and sea spray aerosol. Their results indicated that injecting small Aitken mode particles (in the range 30≤*D*_p_≤50 nm) could result in a reduction rather than an increase in cloud albedo. This is counterintuitive, because one would normally expect that the majority of particles in these size ranges would not significantly reduce the humidity during activation; these particles tend to remain interstitial, owing to the effect of the high curvature of their surface on the equilibrium vapour pressure of water. The effect described by Alterskjaer and Kristjánsson was not seen in earlier studies by Bower *et al.* [9] and Latham *et al.* [8]. Hence, in this paper, we also attempt to reconcile the differences put forward by the aforementioned studies.

## 2. Methodology

We use the same models described by Simpson *et al.* [13]. They are explained briefly here.

### (a) The explicit model

The model used is the aerosol–cloud and precipitation interactions model (ACPIM), a cloud model with bin-microphysics which was developed at the University of Manchester [14]. The aerosol size distribution is input as several lognormally distributed ‘modes’, which are discretized into size bins, each mode requiring the particle number concentration, *N*, the median particle diameter, *D*_m_, and the natural logarithm of geometrical standard deviation, 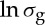. In this study, it is configured so that each mode of the aerosol particle size distribution is split into 200 bins with a minimum size of 5 nm. To best resolve the splitting of the aerosol population upon activation into cloud drops the aerosol bin-widths are configured so that they each contain the same number concentration of particles. The relevant calculation of the albedo is described in Latham *et al.* [8], §4a; however, the difference in this work is that we now use Mie theory to explicitly calculate the extinction efficiency of the aerosols and drops as a function of their size, rather than assume that the extinction efficiency is equal to 2. It is important to use the extinction efficiency calculated from Mie theory in situations where large quantities of small aerosol particles are added (which have a large total projected area, but almost negligible extinction efficiency). The optical depth was calculated by integrating the extinction in the vertical as in equation (4.3) in Latham *et al.* [8]. Although not crucial to the calculation of albedo, we specify whether an aerosol particle is activated into a cloud drop by checking whether it is above the critical diameter, as determined from Köhler theory.

### (b) The parametrizations

To try and reconcile the results put forward by Alterskjaer & Kristjánsson [10] with the results presented by Latham *et al.* [8], we have used two droplet activation schemes to compare with the parcel model. The schemes used are (i) the Abdul-Razzak *et al.* [15] scheme, which was used in the Alterskjaer and Kristjánsson study, and (ii) the Fountoukis & Nenes [16] scheme. Both are quite similar in that they take temperature, *T*, and pressure, *P*, and aerosol properties as an input and output the peak supersaturation and number of activated drops; however, the first scheme uses an approximate analytical solution to find the peak in supersaturation at cloud base, whereas the second method uses an iterative method to find the maximum. The aerosol size distributions are input as lognormally distributed ‘modes’, but are not discretized into size bins, as in the explicit model above.

Mie theory was not used to calculate the extinction for the parametrization methods as there is no information on the size of the unactivated aerosol particles as they swell owing to an increase in humidity. Instead, for these parametrization methods, we assume that the extinction is dominated by the cloud drops and that they have an extinction efficiency of 2. This is a very good approximation for the cloud drops: the explicit model also predicted activated cloud drops to have an extinction efficiency very close to 2. The albedo was calculated from the activated droplet number concentration by assuming that the liquid water mixing ratio increased linearly with height, *q*_l_=*az*, where *z* (m) is height above cloud base. A cloud depth of 160 m was used so that the cloud top liquid water mixing ratio was 0.27 g kg^−1^, which is typical for marine stratocumulus and consistent with the explicit model (§2*a*). These parameters were also chosen so that the cloud albedo *A*_c_∼0.4 in the simulation with no added salt particles (consistent with [17]).

In this case, assuming all particles are the same size, for a given altitude above cloud base the extinction can be calculated by setting the mixing ratio, *q*_l_=(*ρ*_w_*πD*^3^/6)*N*=*az*. Because *q*_l_ is known (by the assumption of an adiabatic liquid water mixing ratio), we then rearrange to find the extinction, *β*=*NπD*^2^/2; hence,
2.1
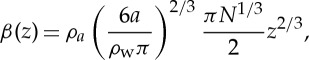

and the optical depth of the cloud is (the vertical integral of equation (2.1))
2.2
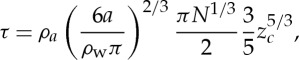

where *z*_c_ (m) is the cloud thickness, *N* (kg^−1^) is the number mixing ratio of cloud drops, *ρ*_w_ is the density of water, *ρ*_a_ is the average density of air for the layer and *a*=1.67×10^−6^ (kg kg^−1^ m^−1^) is a constant. A relationship from Seinfeld & Pandis [18] is used to relate the diurnal mean albedo of a cloud, *A*_c_, to the optical depth via
2.3
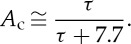



### (c) Model set-up

The aerosol size distributions measured during a recent field campaign that sampled marine stratocumulus clouds off the Chilean coast [19] were used as a basis for the background aerosol size distribution in this study (table 1). We used the ‘remote’ region away from the coast, which used *in situ* measurements of aerosol particles west of 80° W along 20° S. This region is over the Southeast Pacific Ocean, away from the near-coastal polluted regions, and is frequently considered as being suitable for marine cloud brightening in global modelling studies [10,20].

**Table 1.** Background aerosol lognormal fit parameters for the ‘remote’ region in VOCALS (see [19]). Allen *et al.* [19] define the remote region as that west of 80°W. The subscript ‘b’ denotes background aerosol.

	mode 1	mode 2	mode 3
*N*_b_(kg^−1^)	46.64×10^6^	153.42×10^6^	166.77×10^6^
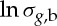	0.348	0.354	0.465
*D*_m,b_(nm)	18	39	154

We performed several sensitivity studies with the model for an updraft speed of 0.3 m s^−1^ and with the injected (salt mode) aerosol having 
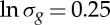
 (narrow) and 0.5 (broad) lognormal distribution parameters. We also varied the median diameter, *D*_m_, of the salt mode using values of 20, 30, 45, 95, 200, 500 and 1000 nm. The total NaCl mass mixing ratio, *q*_salt_, defined as the total mass of NaCl per unit mass of air, was varied by changing the particle number concentration of the lognormal input distribution, *N*. The total NaCl mass mixing ratio, *q*_salt_, was varied between 10^−14^ and 10^−4^ kg kg^−1^ (this equates to 10^−5^ to 10^5^ μg kg^−1^), each subsequent run having mass mixing ratios that were 10 times the previous run.

## 3. Results

### (a) Most efficient sizes/spray technique

The flow rate of water into the atmosphere, *Q*, depends on what fraction of the Earth the scheme is applied to and the *mixing ratio of salt*, *q*_salt_, that is required in that region (appendix B). Because the energy used by the sprayers is proportional to either *Q* (Taylor cone jets, supercritical fluid and effervescent spray) or (*a*/*D*_m_+*b*)*Q* (Rayleigh jets), and *Q* is proportional to *q*_salt_, we chose to plot our results as a function of either the *salt mass mixing ratio* or the *salt mass mixing ratio* multiplied by (0.45/*D*_m_+3.2×10^6^) (see equation (A 8)). This enables an assessment of the most efficient spray distribution for the two different spray techniques by assigning a value of albedo that is approximately 0.05 greater than the baseline case and reading off the graph what the corresponding *x*-axis value is. The *x*-axis value is then multiplied by a constant to scale up to total power required, which depends on the spray technique.

**Figure 1.**
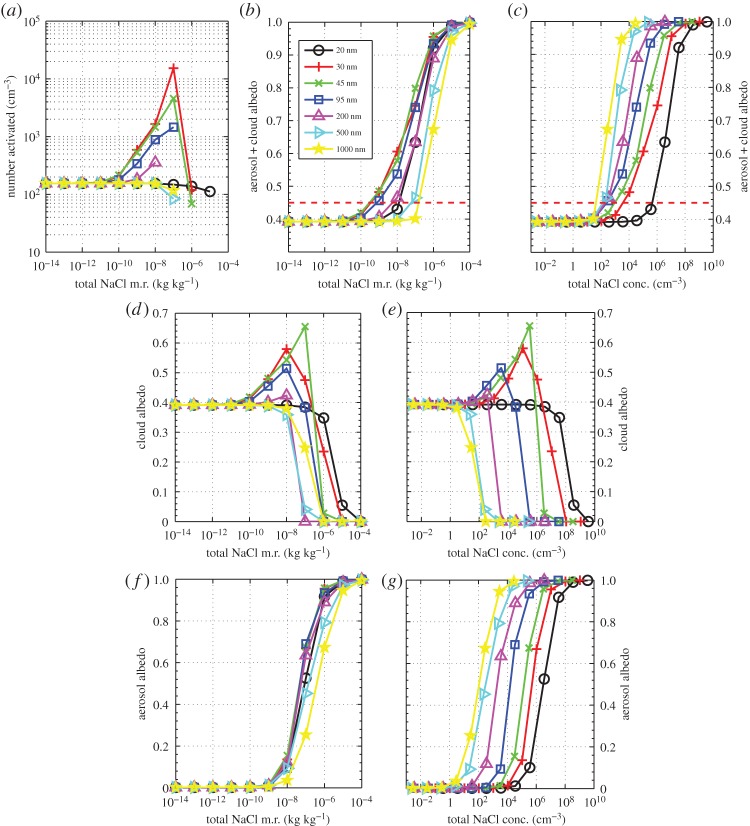
The results from using the ACPIM bin model for different median diameters of the injected aerosol (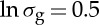, *w*=0.3 m s^−1^). (*a*) The number of activated drops versus the mass mixing ratio of NaCl particles; (*b*) the total albedo versus the mass mixing ratio of NaCl particles; (*c*) the total albedo versus the number concentration of added NaCl particles. Red dashed line is the approximate required value of albedo for geoengineered clouds. (*d,e*) Same as panels *b* and *c*, but just for the cloud particles; (*f*,*g*) same as panels *b* and *c*, but just for the unactivated aerosol particles.

Figure 1 shows results from the ACPIM bin model for an updraft speed of 0.3 m s^−1^ and a 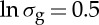. Figure 1*a* shows the number of cloud drops that are formed as a function of *q*_salt_ for different values of median dry diameter of the NaCl particles, *D*_m_. We have plotted the response of the cloud/aerosol particles in different ways. Figure 1*b,c* shows how the albedo of the aerosol and cloud particles changes for different values of *q*_salt_ and NaCl particle concentrations (which ranged between approx. 1×10^−6^ and approx. 10^9^ cm^−3^); figure 1*d,e* shows similar plots but just for the cloud drops (i.e. those particles that grow into cloud drops and do not remain as ‘interstitial’ aerosol particles); and figure 1*f,g* shows similar plots, but just for the interstitial (non-cloud) aerosol particles. Generally, it can be seen that at high values of *q*_salt_ the albedo response is dominated by the interstitial aerosol particles (i.e. the albedo owing to the cloud reduces rapidly for *q*_salt_>10^−6^, but, for aerosols, it increases to values close to unity), whereas at low values of *q*_salt_ it is dominated by the cloud particles (i.e. the albedo owing to aerosols is almost zero for *q*_salt_<10^−10^, but for the cloud it is approx. 0.4).

It is interesting to note that for NaCl mixing ratios that are between 10^−10^ and approximately 10^−7^ the particle sizes that are most effective at changing the albedo of the cloud are particle median dry sizes that are in the range 30≤*D*_p_≤100 nm (figure 1*b*). This suggests that using these particle sizes would be the most efficient way (in terms of least energy consumed) of changing the cloud albedo when using either the *Taylor cone jet*, *supercritical fluid* or the *effervescent spray* methods of spray generation. However, in terms of the number of particles added, it can be seen that the larger particles are the most effective at changing the albedo (figure 1*c*), which is consistent with previous findings. We also see that particle sizes of 30 nm produce the largest cloud drop concentrations (figure 1*a*) and that the dependence of drop concentration on *salt mixing ratios* is monotonic for *mixing ratios* less than approximately 10^−8^ to 10^−7^.

**Figure 2.**
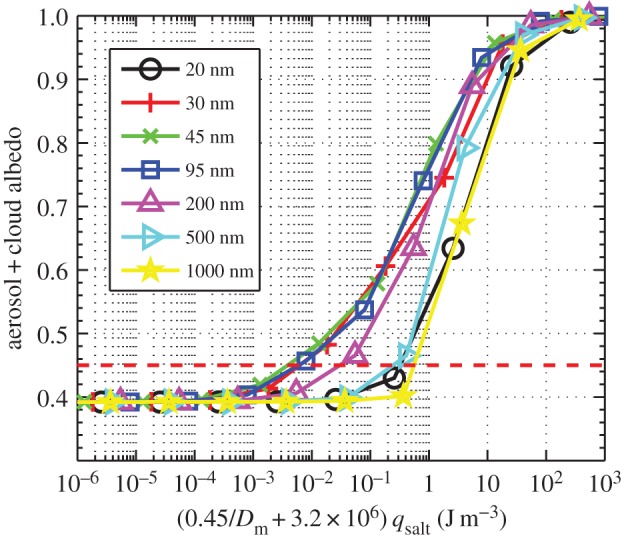
The results from using the ACPIM bin model for different median diameters of the injected aerosol (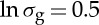, *w*=0.3 m s^−1^). Data are plotted as the albedo versus a variable *χ*=(0.45/*D*_m_+3.2×10^6^)*q*_salt_ (see §1 for details). Red dashed line is the approximate required value of albedo of geoengineered clouds.

Figure 2 shows the same data as figure 1 except that instead of plotting the albedo as a function of total NaCl mixing ratio we have plotted the albedo against a variable, *χ*=(0.45/*D*_m_+3.2×10^6^)*q*_salt_. The reason we have chosen to plot against this variable is because it should be proportional to the energy used by the *Rayleigh jet* method (see equation (A 8), appendix A, §*a*). Figure 2 shows that, for values of *χ* between 10^−3^ and 10^−1^, particle median diameters between 30 and 100 nm are the most effective at changing the albedo. At values of *χ* greater than 10^−1^, the 30 nm particles become less efficient than the 45 and 100 nm particles. The 45 and 100 nm particles are the most efficient at all sizes.

### (b) Comparison of parametrization methods

**Figure 3.**
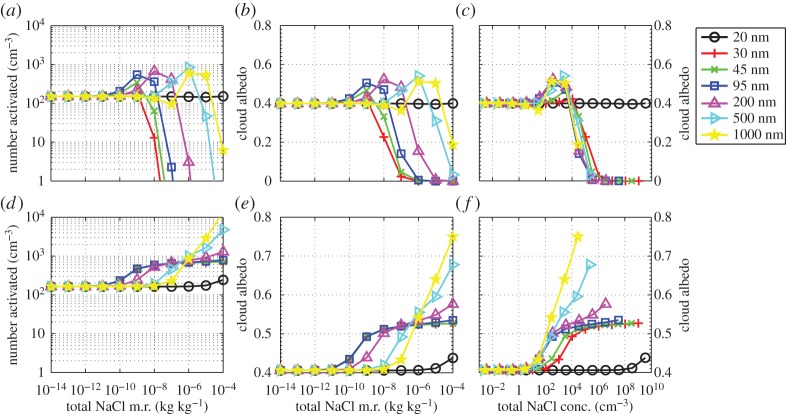
Results from different cloud droplet activation parametrizations. (*a*–*c*) The *Abdul-Razzak et al.* scheme; (*d*–*f*) the *Fountoukis and Nenes* scheme. (*a*,*d*) The number of activated drops versus the mass mixing ratio of NaCl particles; (*b*,*e*) the albedo versus the mass mixing ratio of NaCl particles; (*c*,*f*) the albedo versus the number concentration of added NaCl particles.

Figure 3 shows how the different parametrizations (see §2*b*) behave for an updraft speed of 0.3 m s^−1^ and a 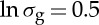 and can be compared with the bin model calculations in figure 1*a*–*c*. It is clearly shown in figure 3*b*–*c* that, for all but the smallest median diameter particles (*D*_m_=20 nm), the Abdul-Razzak *et al.* scheme shows a sharp ‘drop off’ in the predicted albedo as the mixing ratio of NaCl particles (or number concentration) increases. We suspect that this is the region in the aerosol size distribution parameter space where Alterskjaer & Kristjánsson [10] noted a positive radiative effect when adding Aitken particles. This is not evident in the Fountoukis and Nenes scheme (figure 3*e*–*f*).

When comparing the parcel model (figure 1*a*) with the Abdul-Razzak *et al.* scheme (figure 3*a*), we see that the Abdul-Razzak *et al.* scheme underestimates the number of activated drops. This is the reason it underestimates the cloud albedo (figures 1*b* and 3*b*). As pointed out by Simpson *et al.* [13], §3.3, there is too much competition for water vapour in the Abdul-Razzak *et al.* scheme because it assumes the aerosol particles start at their equilibrium size during the activation process, when in fact they need a finite time to grow to these sizes.

The converse is true for the Fountoukis and Nenes scheme, which overpredicts the number of activated drops (cf. figures 1*a* and 3*d*); however, the Fountoukis and Nenes scheme still underestimates the albedo because it does not consider the effect of swollen aerosol on the cloud radiative properties (cf. figures 1*b* and 3*e*). This finding has been discussed by Simpson *et al.* [13], §3.3.

In general, though, the albedo changes caused by adding salt particles are significantly underestimated by both schemes, which is due to the fact that neither of the parametrization methods treats unactivated aerosol in the calculations of albedo. It is evident that both of the parametrization schemes were not designed for the range of inputs that are relevant for geoengineering applications, in which there can be a large quantity of unactivated aerosol particles, and as a result they perform poorly when compared with the parcel model (figure 1*a*–*c*). For the Abdul-Razzak *et al.* scheme, the number of activated particles and hence cloud albedo reduces as *q*_salt_ is increased (figure 3*a*,*b*). This behaviour is qualitatively consistent with the cloud albedo from the parcel model (figure 1*d*); however, the parametrization scheme does not take into account the significance of the large quantities of unactivated swollen aerosol particles.

### (c) Effect of narrowing the spray distribution

**Figure 4.**
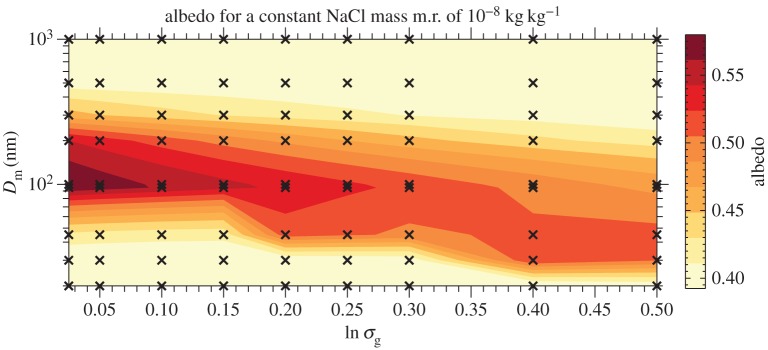
Calculations of the albedo for a constant NaCl mass mixing ratio of 1×10^−8^ kg kg^−1^ as a function of 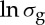 and *D*_m_. This shows that for a narrow distribution the most efficient size is approximately 100 nm, whereas for the broadest distributions the most efficient size is approximately 30 nm.

It should be noted that the width parameter, 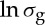, of the injected aerosol also influences the choice of the most efficient spray distribution. To demonstrate the role that 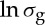 plays we performed additional bin model runs, varying 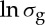 from 0.025 (which is close to the values that the *Rayleigh jet* technique may achieve; appendix A, §*a*) to 0.5 (which is what the *supercritical fluid* technique can achieve; appendix A; §*c*). We did these calculations for a constant mass mixing ratio of salt particles equal to 10^−8^. The results of these calculations are shown in figure 4 and confirm that the most effective size at increasing the albedo increases as the width of the distribution decreases; however, it is evident that, even for the most narrow distribution, the most efficient dry salt particle median diameters are still only approximately 100 nm diameter.

## 4. Discussion

### (a) Optimal spray parameters for each technique

Previous studies of marine cloud brightening have investigated the spray parameters that will be most effective for changing the albedo of the cloud. However, these studies have not considered the spray parameters that are most efficient for a given energy expenditure.

Our model calculations assume an idealized aerosol particle size distribution to test the power needed for marine cloud brightening, assuming no coagulation of the particles. Stuart *et al.* [21] considered the Brownian collection of aerosol particles leaving the spray systems and found that up to 50% of aerosol particles may be removed in neutral to moderately unstable atmospheric conditions that are prevalent in locations of marine stratocumulus clouds. However, Stuart *et al.*'s results suggest that the fraction of aerosol remaining in the atmosphere owing to Brownian coagulation is quite insensitive to the size of the aerosol particles in the range 100<*D*_m_<500 nm. The relative insensitivity to particle size implies that our findings of the most effective particle sizes would remain unaltered.

In determining the most efficient spray particle size for marine cloud brightening, we have considered the four main methods of spray generation: the *Rayleigh jet-instability* or *Taylor cone jet* methods, the *supercritical fluid* method and the *effervescent spray* method. Our results suggest that the spray parameters that are the most efficient for the marine cloud brightening scheme depend on the spray technique used and the amount that the cloud albedo is to be changed by. For all three methods, dry particle median diameters in the range 30≤*D*_m_≤100 nm were the most effective with respect to the power consumption. This suggests that overall the most efficient sizes to aim for are around approximately 100 nm median diameter, which equates to the same volume of an approximately 80 nm sided cube if the salt particles are assumed to be cubes.

The range of aerosol spray parameters investigated is consistent with those achieved by Cooper *et al.* [12,22]; however, the *Rayleigh jet-instability* method may be capable of producing narrower distributions, which would increase the size of the most efficient salt particles (figure 4). Nevertheless, this still implies that median dry diameters of approximately 100 nm or so would be the most efficient.

Offline calculations using physically based parametrizations of cloud activation have been used to understand the implementation of the marine cloud brightening scheme in large-scale models. Our results suggest that these parametrizations do not perform well within the parameter space relevant to marine cloud brightening. The Abdul-Razzak *et al.* [15] scheme, in particular, predicts that large concentrations of Aitken particles lead to a significant reduction in the albedo, which is inconsistent with the parcel model results, mainly because it does not take into account the swelling of unactivated aerosol particles at humidities close to water saturation. Although the Fountoukis & Nenes [16] scheme was generally consistent and qualitatively similar to the parcel model, it also did not quantitatively reproduce the correct range of results, both in terms of number of cloud drops and in terms of albedo.

Alterskjaer & Kristjánsson [10] used the Abdul-Razzak *et al.* [15] scheme within the Norwegian Earth system model to investigate the marine cloud brightening scheme and arrived at the conclusion that the sign of radiative forcing was dependent on both particle size and the mass injected. They found that injecting accumulation mode particles had the desired negative radiative effect, but that injecting large quantities of Aitken mode particles resulted in positive radiative forcing. Such a finding is consistent with figure 3, which highlights that the Abdul-Razzak *et al.* [15] scheme does not perform well in this regime.

### (b) Energy requirements

We now turn to the question of energy requirements for the different spray systems. Latham *et al.* [23, p. 3970] argue that, in order to offset the effects of rising levels of CO_2_, the marine cloud brightening scheme needs to provide a negative radiative forcing of Δ*F*∼−4 W m^−2^ of the Earth's total *F*=340 W m^−2^ average irradiance. Latham *et al.* [23], eqns 3.1–3.3 provide arguments to link the required forcing to a change in cloud albedo. Assuming that the clouds that are to be brightened cover 20% of the Earth's surface the result (from [23] eqn (3.3)) is that the change in cloud albedo has to equal:
4.1
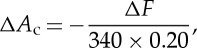

which for Δ*F*=−4 W m^−2^ yields Δ*A*_c_≅0.06; hence, the aim would be to change the albedo of marine stratocumulus clouds by an average of +0.06.

If we consider the Rayleigh jet method we may insert equation (B 1) into equation (A 8) to obtain the power required by the sprayers in terms of the parameter, *χ*=(0.45/*D*_m_+3.2×10^6^)*q*_salt_:
4.2
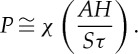

Figure 2 shows that the parameter, *χ* (the value of the *x*-axis), that achieves the required change in albedo for 30≤*D*_m_≤100 nm is approximately 3×10^−3^, so substituting this and other variables into equation (4.2) yields a total power for the Rayleigh jet method of approximately 30 MW, which is a relatively small amount of power—it is comparable to the power consumption of one large ship (40–80 MW).

For the Taylor cone jet method, we may apply equation (A 12), which requires the total flow of water as input. Figure 1*b* shows that an NaCl mixing ratio of approximately 0.5×10^−9^ yields the required change in albedo using dry salt particle median diameters of 30≤*D*_m_≤95 nm, which if substituted into equation (.21) yields the volume flow rate of sea water to be *Q*∼5.5 m^3^ s^−1^. We then estimate the total power required by this technique by substituting *Q*=5.5 m^3^ s^−1^ into equation (A 12), which yields a required power of approximately 6.1×10^3^ MW.

In the supercritical flow spray technique, the NaCl mixing ratio of approximately 0.5×10^−9^ still holds and so *Q*=5.5 m^3^ s^−1^ is substituted into equation (A 15), which yields a required power of approximately 2×10^4^ MW; hence, this method is prohibitively expensive in terms of power consumed by the sprayers. It is approximately 500 times less than current global power generation.

Finally, we consider the power requirement for effervescent spray atomization, which is proportional to *Q* (see equation (A 17)); therefore, the sea water flow rate to aim for is the same as the Taylor cone jet and supercritical fluid techniques: *Q*=5.5 m^3^ *s*^−1^, which is substituted into equation (A 17). This yields a power of approximately 1.9×10^3^ MW; hence, this method is less energy intensive than the Taylor cone jet method, but more intensive than the Rayleigh jet method.

## 5. Conclusion

This paper has investigated the response of marine cloud brightening to the aerosol particle size distribution and addressed two questions: (i) what aerosol size distribution will be most energy efficient to achieve the desired changes in cloud reflectance and (ii) the performance of droplet activation schemes against a more realistic microphysical model. We examined the effect that altering the aerosol particle size distribution has on the activation and growth of drops, i.e. the Twomey effect alone, and have not considered macrophysical cloud responses that may enhance or mitigate the Twomey effect.

Parcel model results:

— in terms of total mass of salt added the most efficient size to seed with is approximately 100 nm median diameter particles in general. However, for the Taylor cone jet, supercritical fluid and effervescent spray systems, it may be just as efficient to seed using particles with a dry median diameter as small as 30 nm; and
— these parcel model results are not in agreement with the recent paper by Alterskjaer & Kristjánsson [10], who found a negative response to the albedo when adding Aitken particles. This study used a parametrized model of activation to determine the number of cloud drops [15,24].

Parametrization results:

— we used two parametrization methods to determine the cloud droplet number concentration for the same conditions as those simulated in the parcel model. They were (i) the Abdul-Razzak *et al.* [15] scheme for lognormal aerosol distributions and (ii) the Fountoukis & Nenes [16] scheme, which is also for lognormal aerosol distributions;
— neither scheme reproduced the dynamical parcel model with high accuracy. In all cases, the Abdul-Razzak *et al.* scheme resulted in a negative response of the cloud droplet number concentration as the total mass of aerosol increased past a threshold that depended on the size of the mode, whereas the Fountoukis & Nenes [16] scheme did not reproduce a negative response. It appears that the reason the parametrization methods do not represent the parcel model results well is that they do not consider the effect of swollen, unactivated aerosol particles on the albedo, which is a significant effect;
— it was found that, in the context of marine cloud brightening, unactivated but swollen aerosol particles have a significant effect on the reflectance/albedo of the cloud layer. Currently, cloud activation parametrizations do not consider this effect; and
— the limitations of the physically based parametrizations should be borne in mind when using them to study aerosol–cloud interactions within large-scale models.

Other:

— there are many other factors that determine what the most efficient spray distribution is. Here, we have merely focused on what we expect the most efficient spray parameters are for a given energy cost. However, factors such as energy availability; maintenance costs; engineering the apparatus to rapidly nebulize the sea spray; and factors that affect the transport of the spray into the cloud base, such as the effect of latent cooling on the buoyancy of the air, may all play an important role;
— our simple calculations of energy usage suggest that the Rayleigh jet method is the least energy intensive, followed by the effervescent spray technique, then the Taylor cone jet method and then the supercritical fluid method;
— the effervescent spray technique assumes that the compression of the gas behind the nozzle results in no loss of heat; however, this assumes that the gas remains at 1000 K, which seems unlikely. Hence, we suggest that more energy would be required for this technique; and
— finally, in order to decide whether such a scheme would be beneficial, a thorough cost–benefit analysis is required.

## Appendix A. Spray methods

**(a) Rayleigh jet/jet instability**

First, there is the technique that makes use of the instability of jets, which was analysed in detail by Rayleigh [25] and is the method proposed by Salter *et al.* [26, p. 3997]. In such a technique, the water jet breaks up into water drops that have a diameter equal to 1.89 times the diameter of the initial cylindrical jet. We now investigate the power requirements for spraying using this kind of jet.

The relationship between pressure drop across a nozzle, Δ*p*, and flow through it, *Q*_s_, is sought. We can interpret the pressure drop across the nozzle to be due to: (i) providing the high kinetic energy required for the jet, Δ*p*_KE_; (ii) capillary pressure owing to surface tension, Δ*p*_ST_; and (iii) viscous flow through the tube, Δ*p*_vis_:

A 1

The pressure drop caused by providing kinetic energy to the drops is

A 2

and it is argued that *v*=80 m s^−1^ is an ideal flow speed to aim for to achieve good drop break-up, which gives Δ*p*_KE_=3.2×10^6^ Pa.

For the capillary pressure, we use the Young–Laplace equation:

A 3

where *σ*=0.072 N m^−1^ (at −20°*C*) and *D*_n_ is the diameter of the end of the nozzle, which results in Δ*p*_ST_≅0.3/*D*_n_ (units are Pa if *D*_n_ is in metres).

Hagen–Poiseuille flow describes the pressure drop, Δ*P*_vis_, in a fluid of viscosity *μ* that is flowing down a pipe of length *L*, diameter *d*_p_. The equation describing the pressure drop is

A 4

where *Q*_s_ is the flow rate down a single nozzle and *L* is the length of the pipe.

The viscous pressure drop across a tapered nozzle, as in Salter *et al.* [27], their fig. 4, can be described by integrating contributions to the pressure drop resulting from elements of the nozzle that obey Hagen–Poiseuille flow. The angle of the taper is 35.26° because it is etched from a crystal. The length of the whole nozzle is 2.4 μm, and the diameter at the untapered end is 1.5 μm. The diameter of the exit hole of the nozzle, *D*_n_, is considered to be variable. This leads to

A 5

where *d*_p_(*x*) is a function describing the diameter of the nozzle along the length of the nozzle, *x*. In order to evaluate the integral, we have to split it into two terms (one describing the 1.5 μm pipe and another describing the taper). A good approximation to this integral is

A 6

With  and *v*=80 m s^−1^, we find that Δ*p*_vis_≅0.60/*D*_n_ (units are Pa if *D*_n_ is in metres).

The power required to pump the liquid through the system is the pressure drop multiplied by the volume flow rate. Hence, we may write down an equation that describes the power (watts) needed for a given flow rate, *Q* (m^3^ s^−1^):

A 7

A 8

The second line is derived from the fact that the drop diameter is approximately two times the nozzle diameter and the drop diameter is approximately four times the dry aerosol diameter, *D*_m_, so *D*_n_≅2*D*_m_.

**(b) Taylor cone jets**

Here, we provide arguments for the application of Hagen–Poiseuille flow to the case of Taylor cone jets and show that it is consistent with measurements by Neukermans *et al.* [11].

The Taylor cone jet method uses an untapered nozzle and does not require the pump to provide drops with a high kinetic energy, because an electric field provides the kinetic energy instead. For an untapered nozzle, the power required to overcome the pressure drop due to viscosity and surface tension is:

A 9

Application of an electric potential to liquid-filled capillary tubes results in an electrospray (see [28]). Cooper *et al.* [22, p. 87] studied the electrospray technique using 16 μm diameter capillaries to generate approximately 100 nm salt cubes from sea water. For a salinity of 35 kg *m*^−3^, 100 nm salt cubes implies that the sea water drop diameters were approximately 750 nm and probably had a geometric standard deviation of . The volume of water in a drop distribution that is lognormally distributed can be calculated via the third moment of a lognormal distribution (http://en.wikipedia.org/wiki/Log-normal_distribution#Arithmetic_moments):

A 10

where *d*=750×10^−9^ m is the drop diameter, *n*=1×10^9^ s^−1^ is the number of particles sprayed per second by a single electrospray and . These values yield *Q*∼5.6×10^−10^ m^3^ s^−1^.

A single electrospray generates *n*=1×10^9^ particles per second [11, p. 513]. The mechanical power, *P*_m_, required to generate the spray from a single nozzle can therefore be estimated using equation (A 9), assuming *L*=1×10^−3^ m; *d*=16×10^−6^ m (the diameter of the capillary), with *Q*∼5.6×10^−10^ m^3^ *s*^−1^ provided by equation (A 10). Neukermans *et al.* [11] then argue that the mechanical power required for a single nozzle should be scaled up by 1×10^8^ for a single application-sized unit with 1×10^8^ nozzles, each with , and that such a unit would use 20 kW of mechanical power. Our calculation assuming Hagen–Poiseuille flow is consistent with this and yields 20.5 kW.

Energy is also required to apply an electrical potential to the nozzle, because a current results by virtue of the movement charged drops [22, p. 85], which Cooper *et al*. argue dominates over the mechanical power requirement. Neukermans *et al.* [11, p. 514] report that the electrical power requirements are 130 kW for a potential of 3.4 kW and current of 1.8 μA. This is for a volume flow rate of 0.33 l s^−1^, which is a conversion factor of (130×10^3^)/(0.33×10^−3^)≅4×10^8^ W m^−3^. It is to be noted that this is less than the theoretical electrical power required (the product of potential difference, *V* , and current, *I*): *V* ×*I*=6.12×10^−3^ watts per nozzle, or 612 kW for the whole system. However, Cooper *et al.* [22, p. 95] provide estimates of 4 mW per nozzle, with a 3 kV potential and 1.33 μA current, which are consistent with the theoretical calculation. The 4 mW per nozzle figure was for a 3.3×10^−12^ *m*^3^ s^−1^ flow rate, which is a conversion factor of 1.2×10^9^ W m^−3^.

Hence using the values that are consistent with theory, the total power required for this technique is

A 11

A 12

In the second line the first two terms on the right-hand side have been neglected as they are vastly outweighed by the third term.

**(c) Supercritical flow spray**

Supercritical flow spray is the technique of heating up the sea water to its supercritical point and then pumping the supercritical fluid through a nozzle [11]. The supercritical fluid is readily transported through the holes as it has almost zero viscosity. However, the technique relies on heating up sea water to the supercritical temperature, which is 373°C. Heating up water to this temperature requires an amount of heat governed by the heat capacity of water and the latent heat of vaporization:

A 13

where *E* is the heat added to the water, *m* is the mass of water, *C* is the specific heat capacity, Δ*T* is the change in temperature that must occur for the ambient sea water to reach the supercritical temperature and *L* is the latent heat of vaporization for water. To continuously heat water to its supercritical point requires the heat to continuously be applied at a power, *P*_w_,

A 14

A 15

where *P*_w_ is the power that must be applied to heat the water at a rate that can meet the required flow of spray into the atmosphere, *Q* (m^3^ s^−1^), and *ρ*_w_ is the density of the water. We find therefore that, for the supercritical fluid method, the mass of sea water sprayed is proportional to the power required by the sprayers.

**(d) Effervescent spraying**

Finally, there is the technique of effervescent spray atomization [12]. This technique involves spraying a mixture of nitrogen and sea water through a nozzle. As the nitrogen exits the nozzle with the sea water, it rapidly expands and breaks up the emerging sea water drops into a fine spray. Cooper *et al*. find that the power required for a flow of 0.41×10^−3^ l *s*^−1^ of sea water is approximately 140 W. Hence, the power required for effervescent spraying scales as

A 16

A 17

Cooper *et al.* [12] state that, for effective spraying, gas-to-liquid mass ratios are approximately 0.345, which for a sea water flow rate of 0.41×10^−3^ l s^−1^, with the gas density equal to 1.1606 kg m^−3^, yields a gas flow rate of 0.122 l s^−1^. They also state that both the liquid and gas need to be compressed to approximately 90 bar. However, the power requirement of this technique is dominated by the power needed to compress the gas. Cooper *et al.* [12] consider this process as isentropic (i.e. *pV* ^*γ*^ is conserved), which requires the gas volume to be compressed to approximately 25 times the original volume.

The work required to compress a volume *V*_1_ of gas at pressure *p*_1_ to volume *V*_2_ and pressure *p*_2_ can be calculated by considering the integral of *pdV* :

A 18

where *γ*=1.4. With *V*_1_/*V*_2_=25, *p*_1_=1×10^5^ Pa, equation (A 18) yields

A 19

In order to calculate the power requirement, we multiply equation (A 19) by the flow rate, 0.122×10^−3^ m^3^ s^−1^, divided by the volume of one compression, *V*_1_; hence, *V*_1_ cancels and we are left with the result that the power to compress the gas for a flow rate of 0.122×10^−3^ m^3^ s^−1^ is 80 W.

We must also consider the power required to move the air through the nozzle at 90 bar. The volume to be pumped through the nozzle during one cycle is one-25th of the original volume, *V*_1_, and the rate at which the volume is pumped out is one-25th of the flow rate, 0.122×10^−3^ m^3^ s^−1^. Hence, the additional power required is the product of the 90 bar pressure and the one-25th of the flow rate into the system, which is equal to 43 W. Thus, the theoretical total power required for a liquid flow of 0.41×10^−3^ l s^−1^ is 80+43=123 W, which approximates well the estimate of 140 W by Cooper *et al.* [12].

Assumption of an isentropic process is not without difficulty: compression of the gas volume to one-25th of the original volume requires that the temperature of the air rises to approximately 1000 K, which is hotter than an internal combustion engine. Hence, the question that arises is whether this could occur without any loss of heat.

## Appendix B. Flow of water

Sea water must be pumped at a flow rate that depends on several variables. If we assume that the area of the globe to be geoengineered is *A*∼1×10^14^ m^2^ (20% of the Earth's surface) and the depth of the boundary layer into which the aerosols are sprayed is *H*∼1000 m, we can calculate the total mass of salt, *M*, that must be sprayed from the salt mass mixing ratio, *q*_salt_:

B 1

where *ρ*_*a*_∼1.2 kg m^−3^ is the density of air. The aerosol particles have a lifetime of around *τ*=3 days in the boundary layer [23], so we can calculate the flux of salt by dividing equation (A 1) by *τ* and by dividing by the salinity of sea water, *S*=35 kg m^−3^, we arrive at the result that the volumetric flux of sea water (m^3^ s^−1^) is proportional to the mass mixing ratio of salt that we require to be present

B 2
